# Older Adult Experiences Pursing Encore Careers in Research at an Age-Friendly University: Longitudinal Evaluation

**DOI:** 10.1093/geroni/igaf122.2501

**Published:** 2025-12-31

**Authors:** Kathryn Nearing, Joanna Fitzgibbons

**Affiliations:** University of Colorado Anschutz Medical Campus, Denver, Colorado, United States; University of Colorado Anschutz Medical Campus, Aurora, Colorado, United States

## Abstract

In 2022, the University of Colorado Anschutz Medical Campus launched a new training program to prepare older adults (average age 68; range: 54- 82) to pursue encore careers in research. The 14-week, 200-hour competency-based training program prepared Older Adult Research Specialists (OARS) for community-facing and participant-centered roles that research study coordinators typically fulfill on study teams: community outreach and engagement, initial screening for eligibility, facilitating informed consent, and addressing social needs that can impede study participation. The overall goal of the training-to-employment pathway program was to increase the age-inclusiveness of the research workforce at CU Anschutz and enhance the capacity of research teams to reach, recruit and retain older adults and other populations in clinical trials. Within 3 years, we trained six cohorts and graduated 78 OARS. This session will feature the longitudinal evaluation of this innovative initiative, focusing on interviews with OARS hired at CU Anschutz and the principal investigators of associated studies. OARS’ key informant interviews were conducted by an independent program evaluator 3-, 6- and 12-months after they were hired. Key themes pertain to OARS’ roles on study teams, longevity in roles, sense of preparedness for role, ongoing needs for professional development, job satisfaction, and OARS’ impact on potential and enrolled study participants, research teams and research studies. This session will also explore the challenges faced by program leaders in advocating for the hiring of OARS, key barriers OARS experienced in the hiring process, and OARS’ key sources of stress and coping in their demanding encore careers.

